# Analysis of blood-induced *Anopheles gambiae* midgut proteins and sexual stage *Plasmodium falciparum* interaction reveals mosquito genes important for malaria transmission

**DOI:** 10.1038/s41598-020-71186-5

**Published:** 2020-08-31

**Authors:** Yingjun Cui, Guodong Niu, Vincent L. Li, Xiaohong Wang, Jun Li

**Affiliations:** Department of Biological Sciences, Biomolecular Sciences Institute, Florida International University, 11200 SW 8th Street, Miami, FL 33199 USA

**Keywords:** Parasite host response, RNAi, Malaria

## Abstract

*Plasmodium* invasion of mosquito midguts is a mandatory step for malaria transmission. The roles of mosquito midgut proteins and parasite interaction during malaria transmission are not clear. This study aims to identify mosquito midgut proteins that interact with and affect *P. falciparum* invasion. Based on gene expression profiles and protein sequences, 76 mosquito secretory proteins that are highly expressed in midguts and up-regulated by blood meals were chosen for analysis. About 61 candidate genes were successfully cloned from *Anopheles gambiae* and expressed in insect cells. ELISA analysis showed that 25 of the insect cell-expressed recombinant mosquito proteins interacted with the *P. falciparum*-infected cell lysates. Indirect immunofluorescence assays confirmed 17 of them interacted with sexual stage parasites significantly stronger than asexual stage parasites. Knockdown assays found that seven candidate genes significantly changed mosquitoes' susceptibility to *P. falciparum*. Four of them (AGAP006268, AGAP002848, AGAP006972, and AGAP002851) played a protective function against parasite invasion, and the other three (AGAP008138, FREP1, and HPX15) facilitated *P. falciparum* transmission to mosquitoes. Notably, AGAP008138 is a unique gene that only exists in *Anopheline* mosquitoes. These gene products are ideal targets to block malaria transmission.

## Introduction

Malaria causes approximately half a million deaths globally each year. Most of the deaths occur in sub-Saharan Africa, and around 95% of deaths are children under the age of five. Although the death rate has dropped by 54% between 2000 and 2013 due to anti-malaria drugs, insecticide-treated nets, and indoor insecticide spraying^1^, malaria control and elimination still face several challenges. At present, vector control strategies are not as effective as it was because of the spread of insecticide-resistant mosquitoes^2^. The control of malaria also faces the challenge of the resistant *Plasmodium falciparum* strains. Therefore, the malaria eradication campaign needs new strategies. Transmission intervention by vaccines, compounds, or transgenic mosquitoes, is a promising approach. However, valid molecular targets for interventions are required.

The malaria causative pathogen, a *Plasmodium* parasite infection in a mosquito, is complicated. Unlike most other pathogens, parasites in host blood are mainly asexual stage parasites in red blood cells, and some develop sexual stage parasites called gametocytes, and only gametocytes can infect mosquitoes^3^. Once a female mosquito takes a meal of infectious blood, gametocytes in blood develop into ookinetes through fertilization in mosquito midguts during the first hours after the ingestion of infectious blood^4^. The blood meal also stimulates the mosquito midguts to secrete proteins and other materials to form a peritrophic matrix (PM) that wraps the blood bolus. PM is visible 4 h after a blood meal^5^. A sexual stage parasite attaches PM and penetrate two physical barriers, the PM and epithelium, to reach the basal lamina, where it develops into an oocyst.

During this infection process, the molecular interaction between mosquito midgut proteins and sexual stage parasites, e.g., gametocytes or ookinetes, directly affects malaria transmission. For instance, alanyl aminopeptidase N 1 (AnAPN1)^6,7^, annexin protein^8^, and fibrinogen-related protein 1 (FREP1)^9^ bind to the sexual stage of *Plasmodium* parasites and facilitate malaria transmission. Antibodies (Ab) against these mosquito midgut proteins prevent *Plasmodium* transmission to *Anopheles*^10,11^. These transmission-blocking targets share some common biochemical features, such as higher abundance in the midgut than in other tissues, the induction of expression immediately after a blood meal^12^, and direct access to large molecules such as Ab in human blood. Based on these features, we chose mosquito genes that are up-regulated three hours after a blood meal, and their products have signal peptides. Then, we examined the interaction between the candidate mosquito proteins and parasites, particularly gametocytes or ookinetes. Finally, we determined the functional relationship between candidate mosquito proteins and *Plasmodium* transmission to mosquitoes. Five novel proteins related to malarial transmission were discovered. These genes and their products are promising targets for transmission-blocking vaccines, drugs, or transgenic mosquitoes for malaria control.

## Results

### Selection of *An. gambiae* proteins that potentially affect *P. falciparum* invasion of midguts

Blood feeding stimulates the midguts to secrete materials to form PM, which surrounds the ingested blood, protecting mosquitoes from potential pathogens in the bloodmeal. The PM is visible in the midgut lumen 4 h after bloodmeal and disappears 24 h after the bloodmeal^5^. The first few hours after ingestion are keys for gametogenesis, ookinete formation, and transmission^4^. Also, gene expression data at 3 h post bloodmeal are publicly available. In order to find mosquito proteins that affect malaria transmission to mosquitoes in midguts, we used four criteria to select candidates: 1) the proteins have signal peptides; 2) the genes express higher in the midgut than in any other tissues (> 1.2-fold); 3) the genes are up-regulated 3 h after a blood meal (> 1.2-fold), and 4) the expression of the genes return to the regular level (naïve mosquitoes) 24 h after a blood meal. Using the four criteria, we selected the proteins containing signal peptides from our previous annotation^13^ and the vectorbase (https://www.vectorbase.org)^14^. We used published oligo-array data^15^, which is also available online at https://vectorbase.org/download/anopheles-gambiaeexpr-statsvb-2019-06txtgz, to determine gene expression. About 76 genes (Table S1) that satisfy the four criteria were chosen as candidates for further analysis. Three previously identified midgut proteins, including FREP1 (AGAP007031), AnAPN1 (AGAP004809), and HPX15 (AGAP013327), are presented in our predicted candidate list (Table S1).

**Figure 1:**
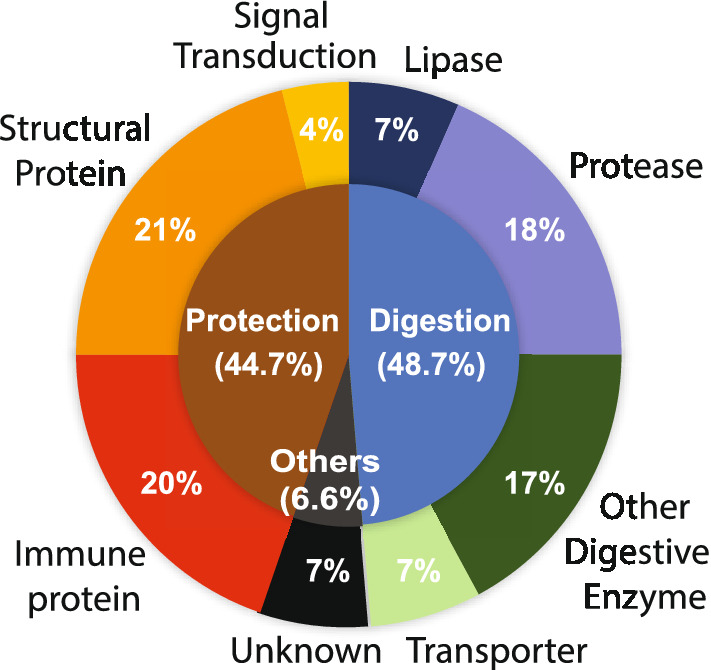
Functional categories of candidate midgut proteins.

These 76 candidate proteins belong to three main functional groups: protection, digestion, and unknown (Fig. 1). About 44.7% of the candidate proteins were expected to protect mosquitoes from infection, which included 15 immune-related proteins (20%), 16 structural proteins (21%), and 3 signal-transduction proteins (Fig. 1). Among these immune-related proteins, HPX15 has been reported to promote malaria transmission^16^. The immune-related proteins also included nine pattern recognition molecules, e.g., peptidoglycan recognition protein, three leucine-rich-repeat (LRR) proteins, and five immunoglobin E-set containing proteins. In terms of the 16 midgut structural proteins, most are involved in the formation of PM, such as chitin-binding proteins, cadherins, and keratin-associate proteins. FREP1, a PM structure protein, has been reported to mediate malaria transmission by interacting with ookinetes^9^.

Moreover, a blood meal triggers midgut cells to secrete enzymes to digest blood^12^. Therefore, a substantial portion (48.7%) of candidate proteins are involved in the digestion process (Fig. 1), which matches the primary function of the insect midgut^17^. Among them, the most common enzymes were peptidases (n = 14), including ten trypsin-like peptidases, two aminopeptidases, and three carboxypeptidases.

### Candidate mosquito midgut proteins that interact with *P. falciparum*

**Figure 2:**
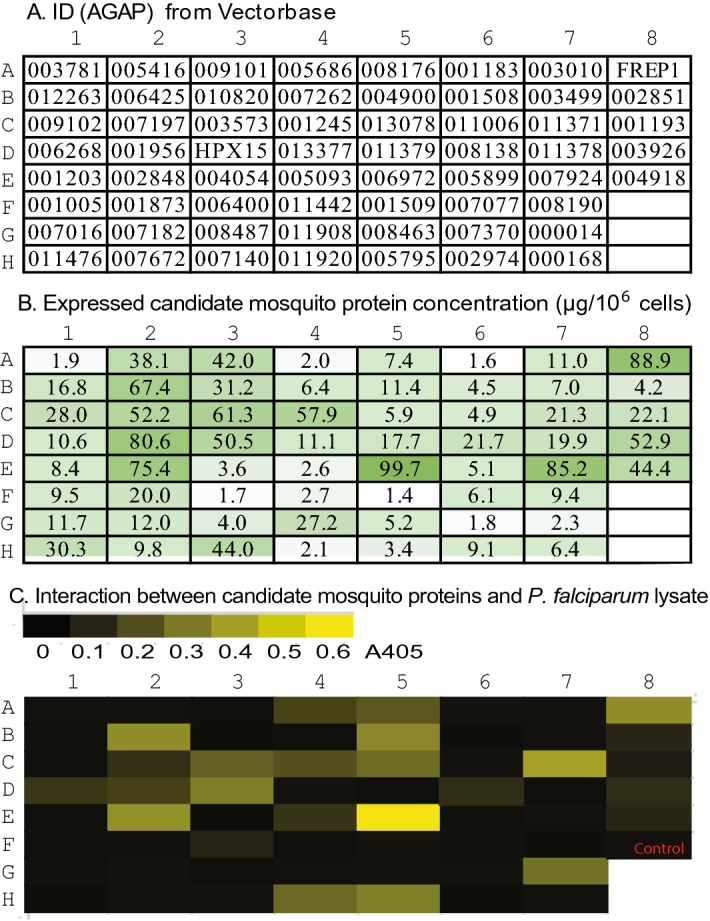
Cloning and expression of the selected midgut proteins and their interaction with *P. falciparum*-infected cell lysates by ELISA assays**.** In total, sixty-one genes were expressed at the detected level. (**A)** Candidate gene IDs. The access number is AGAP(ID)-PA. (**B)** Heterologous expression of the midgut genes with a baculovirus expression system. The color intensity highlights a different expression level for each gene. The concentration of each expressed recombinant protein in each well in panel (**B**) corresponds to the genes at the same position in panel A. (**C)** *P. falciparum*-infected cell lysate was used to coat ELISA plates. The same amount of insect-expressed proteins was added to analyze their interaction with the lysate by standard ELISA. A_405_ values were measured. The signals in panel C correspond to proteins at the same positions in panel A, plus a negative control (heat-inactivated FREP1) at the well of F8. The intensity of the binding signal was represented by the corresponding color: the dark brown color indicated no binding signal was detected, and the yellow color indicated the most substantial binding signal. The binding threshold was that A_405_ of the candidate protein was ≥ threefold over the negative control (heat-inactivated FREP1).

To determine these mosquito proteins that could bind to *Plasmodium* parasites, we cloned and expressed the candidate proteins with a baculovirus system. The amplified gene coding regions were cloned into bacmid and expressed in High Five cells using a serum-free medium to produce the recombinant proteins. FREP1 and HPX15 were expressed in the same system as internal controls. Seven genes, including AnAPN1, were not cloned due to incorrect annotation or other reasons. Eight genes were removed from further study because of their low expression. The remaining sixty-one genes (Fig. 2A) were expressed at the ELISA-detectable level (Fig. 2B). About 23% of the candidate proteins were highly expressed (> 50 μg/10^6^ cells), about 30% (18/61) were moderately expressed (> 10 μg/10^6^ cells), and the rest were expressed at a detectable level (Fig. 2B). Each of the recombinant protein had a 6 × His tag at its C-terminal that would be probed with anti-His monoclonal Ab.

With these 61 insect cell-expressed recombinant proteins, we examined their interaction with *P. falciparum*. The *P. falciparum*-infected cell culture contains cells in various stages: uninfected, asexual stage, and sexual stage parasites (gametocytes and ookinetes). ELISA assays are more sensitive and easier than other methods. Thus, it was used as the screening approach to find candidate proteins that interacted with *P. falciparum*. The 15-day cultured *P. falciparum* that contained more than 10% gametocytes were collected by centrifugation and lysed. The lysate was used to coat the plates, followed by blocking with BSA. The recombinant proteins were diluted, and 0.2 pmol of each recombinant protein was added to each well (Fig. 2A). The retained recombinant proteins in wells were detected by anti-His monoclonal Ab. Recombinant FREP1 (Fig. 2C, A8) and heat-inactivated FREP1 (Fig. 2C, F8) were used as the positive and controls, respectively. The results (Table S2) showed that A_405_ of the 25 proteins were over triple of the negative control (Fig. 2C, Table 1). Out of these 25 proteins, 11 proteins (44%, including FREP1) showed > tenfold of binding signals of the negative control, and 9 proteins (36%) showed medium binding signs (≥ fivefold of the negative control;). It is worth noting that the Histidine tag does not bind to *P. falciparum* lysate based on our previous publication^9^. Indeed, the heat-inactivated 6xHis FREP1 did not show any interaction signal (Fig. 2C, F8). This experiment was conducted twice, and the results were consistent.

**Table 1:** The candidate midgut proteins that interacted with *P. falciparum.*

Idx	ID	AA	Folds	Common name	Biological function	Category
F3	006,400	525	4	Alkaline phosphatase 2	Alkaline phosphatase activity	Digestion
B2	006,425	505	14.1	Cyanogenic beta-glucosidase	Carbohydrate metabolic process	Digestion
C5	013,078	414	10.6	Plasma glutamate carboxypeptidase	Peptide catabolic process, proteolysis	Digestion
D8	003,926	876	5	Aminopeptidase		Digestion
B5	004,900	259	13.4	Trypsin type 4	Serine-type peptidase activity	Digestion
A5	008,176	754	9	Dipeptidyl-peptidase 4		Digestion
A4	005,686	297	7.3	Eupolytin		Digestion
C4	001,245	272	8.3	Eupolytin		Digestion
H4	011,920	255	10.6	Eupolytin		Digestion
H5	005,795	3,499	12.5	Sodium-coupled monocarboxylate transporter 1	Transmembrane transporter activity	Digestion
C3	003,573	814	9.5	Uncharacterized	Bactericidal permeability-increasing protein	Protection
D1	006,268	94	6.1	Peritrophin	Chitin metabolic process	Protection
B8	002,851	151	4.2	Niemann-Pick Type C-2	Intracellular cholesterol transport, innate immunity	Protection
C2	007,197	483	5.4	Niemann-Pick Type C-2		Protection
D2	001,956	152	7	Niemann-Pick Type C-2		Protection
E2	002,848	148	14.3	Niemann-Pick Type C-2		Protection
C7	011,371	666	16.1	Tartan	Leucine-rich repeat protein	Protection
A8	007,031	748	14	FREP1	Pathogen recognition receptor	Protection
E8	004,918	305	4.3	Fibrinogen related protein	Pathogen recognition receptor	Protection
D3	013,327	602	12.4	HPX15	Response to oxidative stress	Protection
E4	005,093	2,175	5.8	Uncharacterized	Cadherin-like superfamily	Unknown
C8	001,193	143	3.1	Uncharacterized	CD59 Glycoprotein	Unknown
D6	008,138	510	4.4	Uncharacterized		Unknown
E5	006,972	113	25.7	Uncharacterized		Unknown
G7	000,014	328	11.1	Uncharacterized		Unknown

Idx: The position in Fig. 2A. ID: Gene access numbers follow the format of AGAP(NUMBER). AA: The number of amino acids. Folds: The folds of A_405_ changes over negative control.

The function of the 25 *P. falciparum*-binding proteins was summarized in Table 1. Ten out of 25 parasite-binding proteins are involved in digestion, including five serine-type peptidases (AGAP004900, AGAP008176, AGAP005686, AGAP001245, and AGAP011920). Ten out of 25 parasite-binding proteins were related to protection, including four niemann-Pick Type C-2 (NPC-2) proteins and three fibrinogen-related proteins (AGAP011371, FREP1, and AGAP004918). Five of 25 parasite-binding proteins were unknown.

### Functional analyses of the candidate *An. gambiae* midgut genes on *P. falciparum* infection in mosquito midguts by RNAi

**Figure 3:**
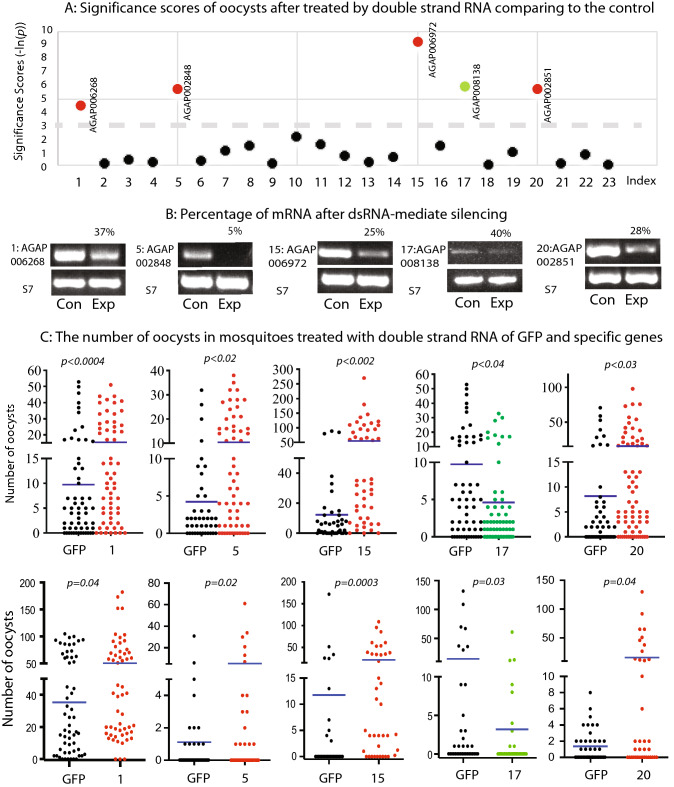
Knockdown effects of *An. gambiae* candidate genes on *P. falciparum* transmission to *An. gambiae*.** (A)** Significance scores of the number of oocysts after dsRNA treatment of a candidate gene compared to the control (score = -ln(p); if a score > 3, the difference is significant). 1: AGAP006268; 2: AGAP006425; 3: AGAP007197; 4: AGAP001956; 5: AGAP002848; 6: AGAP003573; 7: AGAP006400; 8: AGAP005686; 9: AGAP001245; 10: AGAP005093; 11: AGAP011920; 12: AGAP008176; 13: AGAP004900; 14: AGAP013078; 15: AGAP006972; 16: AGAP005795; 17: AGAP008138; 18: AGAP011371; 19: AGAP000014; 20: AGAP002851; 21: AGAP001193; 22: AGAP003926; and 23: AGAP004918. (**B)** The changes in the expression of a candidate gene by quantitative RT-PCR. *An. gambiae* S7 mRNA was used as an internal control. For each gene, the experiments and controls were analyzed in the same gel. We cropped the bands where they showed the gene fragments and S7 fragments to reduce the image size. No other modifications were conducted. (**C)** The number of oocysts in mosquitoes treated with the dsRNA of candidate genes AGAP006268, AGAP002848, AGAP006972, AGAP008138, AGAP002851, and GFP, corresponding to labels 1, 5, 15, 17, 20, and GFP on the X-axis, correspondingly. GFP was used as a negative control. The experiments were repeated twice and showed consistent results. The blue lines indicate the means of the number of oocysts in the midguts.

For the *P. falciparum*-binding midgut proteins, we examined their effects on *P. falciparum* infection in mosquitoes. Because FREP1^12^ and HPX15^18^ were previously examined with knockdown assays, we focused on the remaining 23 candidate genes. Gene-specific dsRNA was injected into the mosquito hemolymph to knock down the expression of a candidate gene. Then we infected the treated mosquitoes with the cultured *P. falciparum*. Seven days after the infection, mosquitoes were dissected to count the oocysts in the mosquito midguts. GFP dsRNA was used as a control. The number of oocysts in each mosquito of the experimental group was compared to that of the control group by a Mann–Whitney-Wilcoxon test. The results are summarized in Table 2 and shown in Fig. 3A. Out of 23 genes, five (AGAP006268, AGAP002848, AGAP006972, AGAP008138, and AGAP002851) showed significant effects on *P. falciparum* transmission to mosquitoes, e.g., the number of oocysts was significantly different between the experimental group and the control group (*p* < 0.05, significance score > 3).

**Table 2:** The data of dsRNA-mediated knockdowns of the tested midgut gene mRNA on *Plasmodium falciparum* infection in mosquitoes.

Index	ID	dsGENE				dsGFP				*P* value	Significance score
		*N*	Mean	SD	Median	*N*	Mean	SD	Median		
1	AGAP006268	60	15.4	14.4	10	58	9.7	13.4	5	0.011	4.53
2	AGAP006425	36	20.8	26	9	23	18.7	19.5	14	0.95	0.05
3	AGAP007197	41	6.4	11.7	2	41	4.2	7.1	2	0.68	0.39
4	AGAP001956	62	20	24.2	10	47	16.3	21.9	8	0.55	0.61
5	AGAP002848	60	10.4	10.5	6	42	4.2	7.1	2	0.003	5.68
6	AGAP003573	44	12.2	25.8	2	50	8.2	16.1	2	0.7	0.36
7	AGAP006400	53	18.2	16.6	12	43	14.5	16.5	10	0.32	1.11
8	AGAP005686	30	11.7	13.2	5	51	14	16.5	7	0.64	0.44
9	AGAP001245	59	33.4	35.2	24	65	32.4	36.2	19	0.91	0.1
10	AGAP005093	52	42.1	38.5	36	65	32.4	36.2	19	0.12	2.08
11	AGAP011920	49	9.9	9.9	7	43	14.5	16.5	10	0.2	1.6
12	AGAP008176	21	26.1	28.3	12	39	38.8	42.3	27	0.31	1.17
13	AGAP004900	39	10.2	12.1	6	58	9.7	13.4	5	0.78	0.24
14	AGAP013078	36	15.6	16.3	12	43	14.5	16.5	10	0.55	0.60
15	AGAP006972	47	55.1	57.5	33	41	12.3	19.5	5	< 0.0001	9.21
16	AGAP005795	52	18.5	25.5	10	41	12.3	19.5	5	0.23	1.47
17	AGAP008138	51	3.9	7.1	1	58	9.7	13.4	5	0.003	5.91
18	AGAP011371	49	15.5	17.9	11	43	14.5	16.5	10	0.97	0.03
19	AGAP000014	53	13.1	17.0	6	51	14	16.5	7	0.37	1.00
20	AGAP002851	69	15.3	21.7	5	50	8.2	16.1	2	0.003	5.71
21	AGAP001193	36	12.8	11.7	12	43	14.5	16.5	10	0.84	0.18
22	AGAP003926	46	7.5	10.2	3	58	9.7	13.4	5	0.47	0.75
23	AGAP004918	33	48	46.6	28	47	50.1	63.1	21	0.85	0.17

The access number of each gene was obtained from Vectorbase.org and followed the format of AGAP(ID)-PA. N: the number of mosquitoes for each treatment. Mean: The average number of oocysts per mosquito; SD: standard deviation; *P* values were calculated using the Mann–Whitney-Wilcoxon test. Significance score equals to –ln(*p*).

If injecting a specific dsRNA into a mosquito did not change the mosquito phenotypes of infection for some reason, such as low knockdown efficiency, we would not analyze it in this report. To determine the knockdown efficiency, we examined the expression level of the candidate genes in the treated mosquitoes using quantitative RT-PCR 48 h after injecting dsRNA. The results confirmed that the expression of five candidate genes was down-regulated by gene-specific dsRNA (Fig. 3B). The expression of AGAP008138 is about 40% of the control, while only 5% of AGAP002848 mRNA was left after dsRNA injection compared to the control. Notably, knocking down either one of AGAP006268 (peritrophin), AGAP002848 (NPC-2), AGAP006972 (keratin-associated protein 16–1), or AGAP002851 (NPC-2) significantly (*p* < 0.05) increased the number of *P. falciparum* oocysts in mosquito midguts compared to the control with 1.6-folds, 2.5-fold, 4.5-fold, and 1.9-fold changes, respectively (Fig. 3C), indicating that these four proteins inhibited malaria infection in mosquitoes. However, knockdown of AGAP008138 (uncharacterized) expression significantly decreased the number of *P. falciparum* oocysts in *An. gambiae*, from 9.7 to 3.9 per midgut (Fig. 3C), suggesting that the AGAP008138 gene product facilitated *Plasmodium* invasion in mosquitoes. The RNAi experiments of the five candidate genes were conducted at least twice, and the results were reproducible (Fig. 3C).

### Confirmation of the insect cell-expressed candidate proteins by western blotting and their interaction with the sexual stage *P. falciparum*

**Figure 4:**
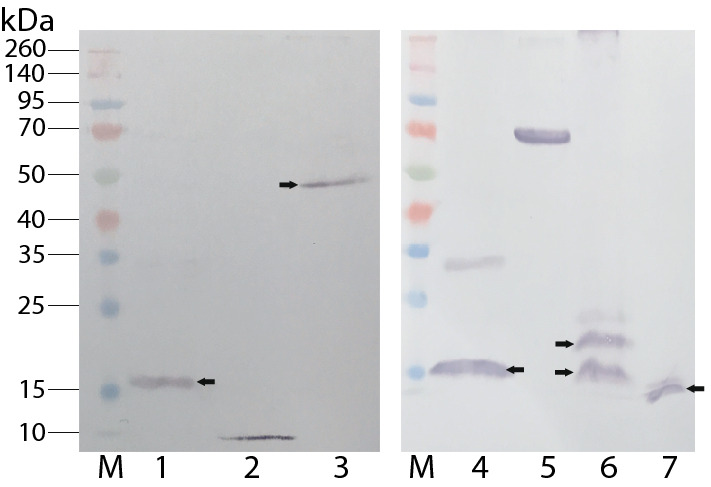
Detection of the insect-cell expressed recombinant proteins by the Western blotting**.** Each recombinant protein contained an additional 15 amino acids, including six Histidine tag at its C-terminus. Lanes: 1, 3, 4, 6, and 7 are AGAP006972, AGAP008138, AGAP002581, AGAP002848, and AGAP006268, respectively. They are the five candidates important for parasite transmission. Arrows point to the precursor or mature forms. Lanes: **M**: protein markers; **2**: AGAP004632; **5**: AGAP013327.

**Figure 5:**
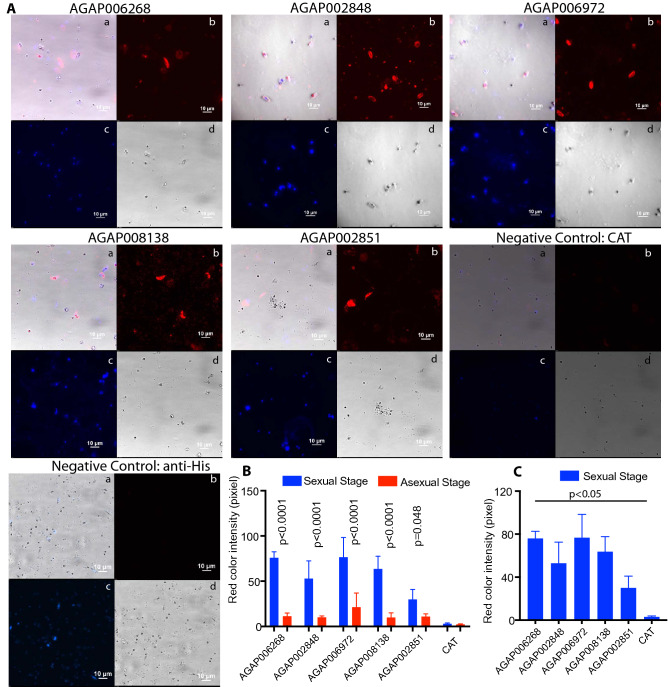
Analysis of a candidate protein interaction with *P. falciparum* sexual parasites by immunofluorescence assays**. (A)** The interaction of the expressed recombinant midgut proteins with *P. falciparum* parasites was confirmed by immunofluorescence assay (IFA). b,c panels show the candidate protein (TRICT) and parasite nuclei (DAPI) respectively; d panel shows the bright views of the cells. Merging column b, c, and d generated a, which shows the co-localization of *P. falciparum* (nuclei) and the midgut protein binding. The banana-shape cells were gametocytes or ookinetes. The round shape cells were trophozoites. The negative control was an unrelated insect cell-expressed protein CAT, which also contained 6xHis tag at its C-terminus. Another negative control just shows that anti-His did not bind cells. (**B)** The graph shows the average fluorescence signal intensity value (+ standard deviations) of three randomly selected regions of sexual parasites (gametocytes and ookinetes) and asexual stage parasites (schizont and trophozoites) as measured with Adobe Photoshop. Statistical calculations by the multiple t test (*p* < 0.05) confirmed the five candidate proteins bound significantly more to sexual stage parasites than to asexual stage parasites. (**C)** The graph shows the average fluorescence signal intensity value (+ standard deviations) of three randomly selected regions of gametocytes and ookinetes in the experimental group and the control. Statistical calculations (*p* < 0.05) confirmed the five candidate proteins bound to sexual stage parasites.

First, we confirmed a set of insect cell-expressed candidate proteins, including the five *P. falciparum*-transmission related proteins, which were specifically recognized by anti-His antibody using Western blotting assays. Samples containing recombinant proteins were separated by 12% SDS-PAGE and analyzed by western blotting assays with anti-His antibodies. Seven recombinant proteins, including the five recombinant proteins, AGAP006972, AGAP008138, AGAP002581, AGAP002848, and AGAP006268, were specifically detected by anti-His monoclonal antibodies (Fig. 4, lanes 1, 3, 4, 6, and 7, respectively). The size of AGAP006972 protein with the signal peptide was predicted about 15 kDa, similar to the observed band (Fig. 4, lane 1). The molecular masses of the mature proteins of AGAP008138 and AGAP002581 were expected to be 55 kDa and 16 kDa, matching the observed bands (Fig. 5, lanes 3 and 4, respectively). The predicted sizes of the precursor and mature AGAP002428 are 18 kDa and 16 kDa, respectively, matching the observed two bands (Fig. 4, lane 6). The size of the AGAP006268 product with the signal peptide was expected to be 12 kDa, matching the observed band (Fig. 4, lane 7). The data supported that the recombinant proteins were expressed by the insect cells in full length with or without signal peptides. This data also demonstrated that the anti-His monoclonal antibody is specific, and the ELISA signals in Fig. 2C corresponded to the bound recombinant proteins.

Next, an indirect fluorescence assay (IFA) was used to examine the interaction between a candidate protein and sexual stage *P. falciparum* parasites, because only sexual stage parasites are relevant to parasite infection in mosquito midguts. Following our previous published methods to generate ookinetes^9,19^, we collected the late-stage cultured *P. falciparum* cells and suspended them in an ookinete culture medium and incubated for 16 h at RT. The cell mixture, containing uninfected cells, asexual stage (trophozoites and schizont), and sexual stage parasites (gametocytes and ookinetes), were deposited onto glass coverslips and fixed with 4% paraformaldehyde. These cells were then incubated sequentially with the insect cell-expressed candidate protein and detected by fluorenes-labeled antibodies. The unrelated insect cell-expressed protein chloramphenicol acetyltransferase (CAT) with 6 × His tag at its C-terminal was used as a negative control. We also added anti-His antibody directly immediately after blocking to confirm the anti-His antibodies did not bind to cells as a negative control. Under a fluorescent microscope, the bound recombinant proteins that were recognized by the anti-His Ab showed a red color, and cell nuclei of parasites appeared blue. Sexual stage and asexual stage parasites were different in shape. The results (Fig S1) showed 24 candidate proteins bound to sexual and asexual *P. falciparum* at various levels. After subtracting the background pixel intensity, we found that all of the ELISA-positive proteins interacted with *P. falciparum*-infected cells (Fig S1A). Out of 24 proteins (FREP1 was excluded), 17 interacted with sexual parasites significantly stronger than asexual stage parasites (Fig S1C). For the five candidate proteins that are related to parasite transmission (Fig. 5A), we found that they significantly bound more to sexual *P. falciparum* parasites than to asexual stage parasites (Fig. 5B, *p* < 0.05). In addition, the interaction signals to sexual stage parasites were also significantly stronger than the negative control (Fig. 5C, *p* < 0.05). It is worth noting that both gametocytes and ookinetes are relevant to malaria transmission. Therefore, we did not distinguish one over the other at this time. Together, our Western blotting data and IFA demonstrated that the five candidate proteins specifically interacted with *Plasmodium* sexual stage parasites (gametocytes and/or ookinetes).

## Discussion

It is essential for *Plasmodium* parasites to infect mosquitoes to complete a malaria transmission cycle. After a blood meal, mosquito midgut epithelial cells secrete materials such as proteins, chitin to form PM, which wraps blood bolus inside. Thus, parasites must overcome the PM first to infect mosquitoes. Only the molecules inside or within PM can contact large molecules such as Ab in the host blood^9^. To identify some midgut proteins that are important for malaria transmission, we focused on proteins that were highly expressed and secreted in midguts and up-regulated three hours post blood meal. We selected these genes as our candidates for further analysis. Consistent with our expectations, functional analysis in our results revealed that most of these candidate proteins were involved in PM formation, microbe recognition, and digestion.

We successfully PCR-cloned and insect cell-expressed > 80% of the candidates. Nearly half (41%) of candidate proteins bound to the cultured *P. falciparum* parasite lysate. The detailed analysis found two known proteins, FREP1 and HPX15, bound to parasites, which was consistent with the previous report^9,16^. They participated in *Plasmodium* transmission to mosquitoes. Seven parasite-binding proteins were peptidases or proteases that should bind to the substrate proteins. Four NPC-2 proteins bound to *P. falciparum* lysates. Two NPC-2 proteins (AGAP002848 and AGAP002851) were immunoglobulin-like secreted proteins, containing MD-2-related lipid-recognition (ML) domain. ML domain is conserved from fungi to plants and animals^20^. NPC-2 proteins play important roles in lipid metabolism and immune signaling pathway^21^. NPC-2 proteins were named because its identification is related to Niemann-Pick disease type C2. In invertebrates, evidence shows that NPC-2 proteins may be involved in innate immune signaling pathways, such as the immune deficiency (Imd) pathway. Three Drosophila NPC2 proteins can bind the bacterial cell wall components of peptidoglycan (PG) and lipoteichoic acid (LTA) to serve as a pathogen recognition receptor (PRR) to initiate the innate immune responses^22–24^. The 25 proteins that bound to the *Plasmodium*-infected cell lysate also included six unknown proteins.

Indeed, all the 25 candidate proteins were confirmed to bind to *P. falciparum*-infected cells by IFA assays. About 68% of these proteins showed significantly higher interaction signals with sexual stage parasites than asexual stage parasites. Since both gametocytes and ookinetes are related to malaria transmission, we did not distinguish them in this report. Among the 25 parasite-binding proteins, 28% (n = 7) significantly affected parasite transmission when the gene expression was knocked down. Two (HPX15 and FREP1) were previously known, and five genes had not been reported as related to *Plasmodium* transmission. By silencing any of AGAP006268 (peritrophin), AGAP006972, AGAP002848 (NPC-2 related), or AGAP002851 (NPC-2 related), the number of *P. falciparum* oocysts in mosquito midguts significantly increased compared to that of the control, suggesting they protected mosquitoes from infection. Specifically, AGAP006268 is a structural protein that is secreted by *Anopheles* midgut epithelial cells after a blood meal to form PM. It contains a chitin-binding domain at its N-terminus^25^, which allows peritrophin to bind to chitin fibers and form the structural basis of PM^26^. Reducing peritrophin expression might destroy the integrity of PM, and facilitates parasite penetration of PM. The function of AGAP006972 is unknown. There are several repeats in the 96-amino acid AGAP006972 peptide, including eight GGY repeats and three GGF. The two NPC-2 proteins (AGAP002848 and AGAP002851) may serve as a PRR to trigger the innate immune response.

Different from the other four genes, but similar to FREP1, knocking down the expression of AGAP008138 significantly reduced the number of *P. falciparum* oocysts in the mosquito midguts. The mature AGAP008138 has 492 amino acids in length. However, AGAP008138 does not have any known motifs. Its expression was induced 7.5-fold 3 h post-blood meal and decreased close to or less than naive mosquitoes after 24 h. IFA showed that it bound to ookinetes. Notably, AGAP008138 is a unique gene, existing only in the *Anopheles* species and not in *Aedes* or *Culex,* which is consistent with the fact that only *Anopheles* mosquitoes transmit malaria. Therefore, AGAP008138 might be an ideal target for malaria transmission-blocking vaccine development, although the detailed mechanisms of these mosquito genes on parasite transmission need further investigation.

This study identified a set of new midgut proteins that interact with *P. falciparum* ookinetes and affect malaria transmission. Further investigation of these proteins will help to elucidate the molecular mechanisms of a *Plasmodium* invasion in mosquito midguts as well as the innate immunity in mosquitoes.

## Materials and methods

### Rear mosquitoes

*An. gambiae* (G3 strain) eggs were obtained from BEI Resources (https://www.beiresources.org/). The insectary was set at 27 °C, 80% relative humidity, 12-day/night cycles. Larvae were fed with grounded fish food, and adult mosquitoes were maintained with 8% sucrose solution.

### Culture *P. falciparum* gametocytes and ookinetes

*P. falciparum* (NF54) was obtained from BEI Resources, and cultured with RPMI-1640 complete medium, containing 4% new O^+^ human red blood cells, 10% human AB + serum, and 12.5 μg/mL of hypoxanthine in a candle-jar at 37 °C. The culture started at 0.1% parasitemia, and day 15–17 *P. falciparum* cultures were used to infect the mosquitoes or to be lysed, as described previously^9^. To prepare *P. falciparum* ookinetes, we transferred 5 mL of day-15 cultured *P. falciparum* containing ~ 2% stage V gametocytes into a 15 mL centrifuge tube and centrifuged at room temperature at 650 g for 5 min (m). The pellet was washed with RPMI-1640 three times and then resuspended in 500 µL of sterile ookinete culture medium (RPMI-1640, 20% human serum AB^+^, 50 µg/mL of hypoxanthine, 2 g/L NaHCO_3_). The resuspended cells were transferred into a well of a 12-well plate and incubated at room temperature on a shaker (50 rpm) for 24 h to generate ookinetes. Finally, the cell mixtures of the ookinetes, gametocytes and the asexual-stage *P. falciparum* were collected by centrifugation at 650 g for 5 m at RT.

### Selection of candidate *An. gambiae* midgut proteins

We focused on the proteins that may contact parasites directly in mosquito midgut lumen. PM starts to form inside of the midgut from secretory materials immediately after a blood meal, reaching its thickest 18 h post blood meal. Therefore, we chose the proteins that have signal peptides, and the corresponding genes are immediately up-regulated by a blood meal, return to regular expression at 24 h after a blood meal, and expressed higher in midguts than in other tissues. The candidate *An. gambiae* midgut proteins were selected from the databases of ReAnoXcel^13^, vectorbase^14^, and AgExpression^15^ according to the following four criteria: (1) candidate proteins contain N-terminal signal peptides based on *An. gambiae* protein databases from previous annotation^13^ and the vectorbase (https://www.vectorbase.org)^14^ ; (2) the expression of candidate genes is > 1.2-fold higher 3 h post bloodmeal comparing to naïve mosquitoes; (3) the expression of candidate genes should return to the expression level of naïve mosquitoes 24 h post-bloodmeal; and (4) candidate genes are expressed > 1.2-fold higher in midguts than in other tissues. The gene expression data were obtained from the vectorbase database (https://vectorbase.org/download/anopheles-gambiaeexpr-statsvb-2019-06txtgz) and AgExpression of a previous publication^15^. The protein functions were obtained from our previous annotation^13^ and the uniprot database (https://www.uniprot.org).

### Cloning and expression of candidate genes in insect cells

Total RNA was extracted from adult female mosquito midguts using RNAzol (Sigma-Aldrich, MO). About 20 midguts were dissected from three-day-old female *An.* gambiae and put into a 1.5 mL plastic tube that contained 200 μl of RNAzol. The tissue was grounded by a micro pestle (Sigma-Aldrich). Eight hundred µL RNAzol was added. After centrifugation (13,000 g for 10 m), RNA in the supernatant was precipitated by isopropanol (1:1 v/v). The cDNA was synthesized with the Superscript First-Strand Synthesis System (Invitrogen, CA). Gene fragments were amplified by PCR using the DNA Engine Dyad Thermal Cycler (Bio-Rad, CA) with gene-specific primers (Table S3). DNA fragments were purified through a GeneJet PCR Purification Kit (Thermo Fisher Scientific) and cloned into modified donor plasmid pFastBac1 that contains a 6 × His tag at the C-terminal of multiple cloning sites. Recombinant plasmids were transformed into competent DH5α cells. Positive recombinant plasmids were confirmed by sequencing and then transformed into DH10Bac to obtain recombinant Bacmids that showed white colonies on culture plates. The positive white colonies were picked and confirmed by PCR. One μg of a recombinant Bacmid in 100 μL un-supplemented grace medium (Invitrogen) was mixed with 100 μL of un-supplemented Gibico grace medium (Thermal Fisher Scientific), containing 5 μl of cellfection II (Thermal Fisher Sci), and incubated for 30 m at RT. The mixture was then added into 1.5 mL of unsupplemented grace medium that contained 800,000 sf9 cells in a well of a six-well culture plate and incubated for 4 h at 26 °C. The supernatant was replaced with 2 mL of complete race medium with 10% FBS (Invitrogen) and incubated at 26 ºC for three days to generate recombinant baculovirus viruses. A 100 μL culture supernatant that contained the recombinant virions were added in 2 mL of Express Five SFM medium (Gibco) supplemented with 20 mM of L-glutamine and 1 million High Five cells in a well of a six-well culture plate. The cells were incubated at 26 ºC for three days to express the recombinant proteins of interest. The recombinant proteins were quantified with anti-His monoclonal Ab using ELISA plates twice.

### Analysis of the interaction between a recombinant protein and the *P. falciparum* lysate using ELISA

Day 15 cultured *P. falciparum* (containing > 10% stage IV and V gametocytes) were harvested by centrifugation (300 g for 5 m). The cells were lysed in native cell lysis buffer (Clontech) and ultra-sonication for 2 m on ice, pausing for 50 s (s) every 10 s of sonication. Cell debris was removed by centrifugation (13,000 g for 5 m), and the protein concentration in the supernatant was measured with the Bradford method. About 50 µL of 3 µg/mL *P. falciparum* parasite protein was added per well on a 96-well plate and incubated at 4 ºC overnight. The plate was blocked with 100 µL of 2% BSA for 2 h at room temperature; then, 0.2 pM recombinant candidate proteins in 50 µL PBS were added to each well and incubated for 1 h. Then, 50 µL of anti-His mouse monoclonal Ab (Sigma, 1:3,000 dilution with blocking buffer) was added to detect the recombinant protein and incubate at RT for 1 h, followed by incubation with 50 µL of goat anti-mouse IgG-Alkaline Phosphatase conjugate (Sigma, 1:10,000 dilution in blocking buffer ) for 1 h. The plate was washed three times with PBST between incubations. Finally, 50 µL of *p*-nitrophenyl phosphate (pNPP) (Sigma-Aldrich, MO) was added for development, and the A_405_ was measured. The insect cell-expressed FREP1 recombinant protein was incubated for 30 m at 65 °C to inactivate FREP1. The heat-inactivated FREP1 was used as a negative control because it did not bind to the parasites, as reported previously^19^.

### Determination of the effects of *An. gambiae* genes on *P. falciparum* transmission to mosquitoes using dsRNA mediated gene expression silencing assays

The procedure was similar to the previous report^27^. Gene-specific primers that contained a T7 promoter and candidate gene sequences were designed with the E-RNAi web server^28^. Double-stranded RNAs (dsRNA) were synthesized with a MEGAscript Kit (Thermo Fisher Scientific, MA). GFP dsRNA was used as a control. About 67 nl of 3 µg/µL dsRNA was injected into 1-day old *An. gambiae* hemocoel using Nanoject II (Drummond Scientific, PA), and 100 mosquitoes were injected for each candidate gene. Thirty-six hours after treatment, the treated mosquitoes were fed with 0.2% *P. falciparum* gametocytaemia. The midguts of the infected mosquitoes were dissected seven days post-infection and stained with 0.1% mercury dibromofluorescein disodium in PBS. Oocysts were counted under a light microscope. The Mann–Whitney-Wilcoxon test was used to determine the statistical difference of the oocysts between the experimental group and the control. The transcript knockdown efficiency was confirmed by the quantitative RT-PCR in five of treated mosquitoes that were taken randomly 24 h after infection. GelDoc (UVP, Upland, CA) was used to take photos of the gels. PCR reactions (to detect specific genes and loading control S7) from one sample were run in the same gel. The bands from a particular gene and S7 were cropped for easier interpretation and smaller file sizes.

### Detection of recombinant proteins with a monoclonal anti-His antibody with Western Blotting

To maximize the yield of recombinant proteins, we collected the expressed proteins in both culture medium and cells. The culture cell medium was separated by centrifugation (650 g for 4 m). The supernatant was concentrated 100-fold by Centricon 10 Centrifugal Filter Device (Millipore, Danvers, MA). The cell pellets were lysed in native cell lysis buffer (Clontech). The cell lysis and concentrated medium were mixed and loaded on 12% SDS-PAGE and separated by electrophoresis until the Bromophenol blue reached the bottom. The proteins on the gel were then transferred on nitrocellulose membrane. The membrane was blocked with 2% BSA in PBST, followed by incubation in mouse monoclonal anti-His antibody (Sigma, 1:2000 dilution with blocking buffer) at RT for 1 h, goat anti-mouse IgG-alkaline phosphatase conjugate (Sigma, 1:10,000 dilution in blocking buffer ) for 1 h. The membrane was washed three times with PBST between incubations. Finally, nitroblue tetrazolium (Sigma-Aldrich, MO) was added to develop.

### Indirect immunofluorescence assays to determine midgut protein-ookinete interactions

The cultured *P. falciparum* gametocytes with ookinetes were deposited onto premium cover glass slips to make blood smears. Before the smears were completely dry, the semi-dry smears were fixed in 4% paraformaldehyde in PBS for 30 m at RT to keep the cell membrane intact. Then, the cover glass slips were sequentially incubated in 1 mL of PBS that contained 10 mM glycine for 20 m and the blocking buffer for 1.5 h at RT. After blocking, cells on a coverslip were incubated with a candidate recombinant protein (10 μg/mL) in 200 μl of PBS containing 0.2% BSA (blocking buffer) for 1 h, followed by sequential incubation with 4 drops of enhancer (Alexa Fluor ® 594 Goat Anti-mouse SFX kit, Invitrogen) for 30 m, 200 μl of anti-His monoclonal Ab (1:2,500 dilution in the blocking buffer, 2 μg/mL) for 1 h, and 200 μl of secondary Ab (Alexa Fluor ® 594 Goat Anti-mouse SFX kit, Invitrogen; 1:1,000 dilution in the blocking buffer) for 30 m. Between each incubation, the slip was washed three times with 1 mL of blocking buffer for 3 m each. In the end, the coverslip was rinsed in distilled water for 20 s, and then coated with 4,6-diamidino-2-phenylindole (DAPI; Sigma-Aldrich). About 20 μl of VECTASHIELD anti-fade mounting media (Vector Laboratories, Burlingame, CA) was added onto the coverslip and mounted onto a slide. After incubation for at least 2 h in the dark, the cells were examined under fluorescence microscopy (Nikon Eclipse Ti-S fluorescence microscope). An unrelated protein chloramphenicol acetyltransferase was used to replace a candidate protein as a negative control, as demonstrated previously^19^. We also added anti-His antibody directly immediately after blocking to confirm the anti-His antibodies did not bind to cells as a negative control. To quantify the binding proteins, we measured the red pixel intensity values of the parasites at three randomly selected regions using Adobe Photoshop (version 2018, San Jose, CA). All values subtracted the background value. Then, we calculated the difference of the mean red pixel intensity values between sexual parasites and asexual parasites or the negative control and experimental groups.

## Supplementary information

Supplementary information.

Supplementary Table S1.

Supplementary Table S2.

Supplementary Table S3.

## Data Availability

All data are available from public databases or this manuscript.
