# Supplementary material for: Analysis of blood-induced Anopheles gambiae midgut proteins and sexual stage Plasmodium falciparum interaction reveals mosquito genes important for malaria transmission
Source: Sci Rep. 2020 Aug 31;10:14316. doi: 10.1038/s41598-020-71186-5 (PMC7459308; doi:10.1038/s41598-020-71186-5)
Supplement: Supplementary file 1 — Supplementary information. [file 41598_2020_71186_MOESM1_ESM.docx]

**Analysis of blood-induced *Anopheles gambiae* midgut proteins and sexual stage *Plasmodium falciparum* interaction reveals mosquito genes important for malaria transmission**

Yingjun Cui, Guodong Niu, Vincent L. Li, Xiaohong Wang, and Jun Li†

Department of Biological Sciences, Biomolecular Sciences Institute, Florida International University, Miami, Florida, USA 33199

† Corresponding author:

Dr. Jun Li

Department of Biological Sciences

Florida International University

OE 214, 11200 SW 8th Street, Miami, USA

Tel: 305-348-7618

Email: [lij@fiu.edu](mailto:lij@fiu.edu)

**Figure S1: Candidate mosquito midgut proteins bound to *P. falciparum*-infected cell**.

A: Insect cell-expressed candidate mosquito midgut proteins could bind to *P. falciparum*-infected cells (red color). The banana-shape cells were gametocytes and ookinetes. The round shape cells are trophozoites. The signals from sexual stage parasites were statistically compared to that from the asexual stage parasites by multiple t-tests. ***: p<0.0001. **: p<0.001; *: p<0.05. B: Negative controls. The insect-cell expressed chloramphenicol acetyltransferase (CAT) was used to substitute a candidate protein and performed the same test. We also used the anti-His antibody directly without adding any other proteins and performed the same IFA assays. C: Quantitative analyses of the interaction between a midgut protein and parasites.
